# Economic Impacts of Ultrasonographic Fetal Sex Determination on Hanwoo Cattle Profitability and Market Dynamics

**DOI:** 10.3390/vetsci12030201

**Published:** 2025-02-27

**Authors:** Doyoon Kim, Miyeon Son, Daejin Jung, Seongeun Heo, Myoungok Kim, Junkoo Yi

**Affiliations:** 1 Department of Animal Science and Biotechnology, Research Institute for Innovative Animal Science, Kyungpook National University, Sangju 37224, Republic of Korea; kdy51311@korea.kr; 2 Gyeongsangbukdo Livestock Research Institute, Yeongju 36052, Republic of Korea; djhoop@korea.kr (D.J.); hse7451@korea.kr (S.H.); 3 Department of Agricultural Economics, Purdue University, West Lafayette, IN 47907, USA; son89@purdue.edu; 4 School of Animal Life Convergence Science, Hankyong National University, Anseong 17579, Republic of Korea; 5 Gyeonggi Regional Research Center, Hankyong National University, Anseong 17579, Republic of Korea

**Keywords:** breeding profitability, economic analysis, fetal sex determination, Hanwoo, ultrasonography

## Abstract

(1) Background: The Hanwoo cattle industry, valued for its premium beef, faces economic challenges due to fluctuating market prices, rising production costs, and profitability disparities based on calf sex, with male calves fetching higher prices and creating income imbalances for breeding farms. (2) Methods: This study assessed the economic impact of ultrasonographic fetal sex determination, a technology enabling fetal sex identification between 55 and 100 days of gestation, using 107 pregnant Hanwoo cows, of which 104 were successfully assessed with a 96.1% accuracy rate across all gestational stages. (3) Results: Economic analysis revealed that farms employing this technology achieved higher profitability by adjusting prices based on calf sex, with male calves significantly enhancing asset value; however, the technology’s financial benefits were influenced by market dynamics, implementation costs, and sex ratios. (4) Conclusions: These findings underscore the potential of ultrasonographic fetal sex determination to improve Hanwoo farm profitability and sustainability, provided its adoption is accompanied by careful consideration of costs and market conditions.

## Simple Summary

The Hanwoo cattle industry faces economic challenges due to market fluctuations and calf sex-based profitability gaps. Male calves, preferred for meat production, increase farm asset values by USD 760. Ultrasonographic fetal sex determination, achieving 96.2% accuracy, enables strategic breeding, price adjustments, and cost savings for farms. Despite initial costs, its long-term benefits improve profitability, market efficiency, and sustainability for Hanwoo farmers.

## 1. Introduction

The Hanwoo cattle industry plays a pivotal role in South Korea’s agricultural sector, with Hanwoo beef being highly esteemed for its distinctive quality and flavor. However, farmers encounter several economic challenges, primarily due to fluctuating market prices, rising production costs, and significant differences in profitability based on the sex of calves [1,2,3,4]. Unlike other globally recognized cattle breeds, Hanwoo is native to South Korea and is highly valued for its marbled beef quality. However, its production is challenged by specific market conditions, calf-sex-related profitability gaps, and the physiological characteristics of Hanwoo cows, such as higher fat deposition, which impacts fetal imaging during ultrasonography. To the best of our knowledge, this study is the first to assess the economic impact of ultrasonographic fetal sex determination in Hanwoo cattle, offering actionable insights for this unique farming context. In particular, the profitability of the breeding sector is significantly influenced by calf sex [5]. Male calves are preferred for meat production and fetch higher prices, while female calves are sold for relatively lower prices, which has a considerable impact on farm income [6,7].

Hanwoo cattle, also known as Korean native beef cattle, are renowned for their high marbling, tenderness, and unique flavor, which make Hanwoo beef a premium product among consumers. Over centuries, Hanwoo cattle have adapted to South Korea’s mountainous terrain and varying climate, resulting in a breed with robust physiology and adaptability. In South Korea, Hanwoo farming is typically conducted on small- to medium-sized family-owned farms, with an average herd size of 10–50 cattle per farm. Hanwoo cattle not only contribute to the economy but also hold cultural significance, as their beef is associated with traditional celebrations and family gatherings. However, challenges such as high production costs and reliance on imported feed have heightened the need for strategic management practices to enhance farm profitability and sustainability.

The Hanwoo farming industry is typically divided into three main operational categories: breeding, fattening, and integrated operations, which encompass both breeding and fattening. Breeding farms, primarily engaged in the production and sale of calves, often have lower operating costs. However, the profitability of these farms is largely determined by the sex of the calf. The average price for male calves is approximately USD 2370, while female calves are sold for around USD 1610, resulting in a significant discrepancy in income. This disparity can place a substantial financial burden on farmers, particularly those engaged in breeding operations, and poses a long-term risk to the sustainability of the Hanwoo breeding industry [8].

To address these challenges, ultrasonographic fetal sex determination technology has gained attention as a promising solution. This technology allows farmers to accurately determine the sex of a fetus between 55 and 100 days of gestation, enabling them to optimize their breeding strategies and maximize profits. Farmers may choose to raise a cow carrying a male fetus for meat production purposes rather than raising the calf itself. Alternatively, if sufficient replacement animals are available, they could sell a heifer or a cow carrying a female fetus to optimize their operational efficiency and income.

In livestock management, the preference for calf sex varies depending on the production goals of the farm. Farms focused on meat production prefer male calves, while breeding farms place higher value on female calves. Therefore, knowing the sex of the calf before birth provides a significant advantage, allowing farmers to plan their breeding strategies 6 to 7 months in advance. Additionally, fetal sex determination offers important benefits in biotechnology research, particularly in improving artificial insemination and embryo transfer programs [9,10,11].

Current research on fetal sex determination includes methods such as sperm sorting [12,13,14,15], embryo sexing [16,17,18] and post-conception techniques like amniocentesis [19,20] or ultrasonography [21,22,23]. Sperm sorting, which involves separating X- and Y-chromosome-bearing spermatozoa, is a well-established method for pre-determining the sex of offspring before conception. This technique has been employed in cattle breeding to significantly increase the likelihood of producing calves of the desired sex, particularly in herds prioritizing male calves for meat production or female calves for breeding purposes. However, while sperm sorting offers strategic advantages, its application in Hanwoo cattle remains limited due to challenges such as reduced conception rates compared to conventional artificial insemination, high costs associated with the sorting process, and the need for specialized equipment and technical expertise.

Embryo sexing, which involves biopsy or molecular analysis of pre-implantation embryos, is another method for fetal sex determination. Although highly accurate, this technique requires laboratory facilities, is invasive, and is often cost-prohibitive for routine use in commercial operations. Similarly, amniocentesis, an invasive method performed during pregnancy to analyze fetal cells for sex determination, poses risks to both the fetus and the dam, limiting its widespread use. In the context of Hanwoo cattle, sexed sperm has been explored as an alternative or complementary approach to fetal sex determination. This method involves sorting spermatozoa based on the X or Y chromosome and can significantly increase the likelihood of producing offspring of the desired sex. For farms prioritizing male calves for meat production or female calves for breeding purposes, sexed sperm could offer a strategic advantage. However, the application of this technology in Hanwoo cattle is limited by relatively low conception rates compared to conventional artificial insemination, higher costs, and technical challenges associated with sperm sorting procedures. Despite these limitations, combining the use of sexed sperm with ultrasonographic fetal sex determination could provide a more comprehensive breeding strategy, optimizing both economic and operational outcomes for Hanwoo farmers. Among these, ultrasonographic fetal sexing is particularly advantageous because it is easily applied under field conditions and provides rapid results [24]. This technique visually identifies the fetus’s scrotum, mammary gland tissues, or genital protrusions (e.g., penis, prepuce, vulva, clitoris) within the uterus [23,25]. Unlike the aforementioned methods, ultrasonography is non-invasive, cost-effective, and provides real-time results, making it highly suitable for routine application in Hanwoo cattle farms. Furthermore, combining ultrasonography with other advanced technologies, such as sexed sperm, could optimize breeding strategies by aligning pre-conception and post-conception sex determination techniques. This integrative approach has the potential to maximize economic returns while addressing the operational needs of Hanwoo farmers.

The optimal timeframe for observing fetal genitalia is between 55 and 60 days of gestation [26,27]. Conversely, the most precise identification of the scrotum and mammary glands occurs between 73 and 120 days [28]. Ultrasonography can determine the fetal sex with 96.3% accuracy between 60 and 90 days of gestation [28,29,30,31]. Furthermore, ultrasonographic sexing necessitates a brief examination period, with an average of two minutes required to ascertain the fetal sex [23].

The implementation of ultrasonographic fetal sexing technology has the potential to markedly enhance the sustainability of the Korean Hanwoo industry. This technology can be employed by farmers to facilitate strategic planning of breeding programs and the formulation of more informed economic decisions. By acquiring knowledge of fetal sex at an early stage, farmers can reduce unnecessary expenditure, optimize production in accordance with market demand, and enhance overall farm efficiency. This, in turn, contributes to increased farm profitability and strengthens the economic stability of the entire Hanwoo beef cattle industry.

The objective of this study is to evaluate the economic impact of ultrasonographic fetal sexing technology on the profitability of Hanwoo breeding farms in Korea. By analyzing the long-term benefits of this technology, the research provides insights into how Hanwoo farmers can leverage it to improve their operations and drive positive changes across the broader Hanwoo beef cattle industry.

## 2. Materials and Methods

### 2.1. Animals

All procedures involving animals in this study were conducted in accordance with the relevant national legislation and guidelines for the care and use of animals. The study was approved by the Institutional Animal Care and Use Committee of the Gyeongsangbukdo Livestock Research Institute (Approval No. GAEC/161/23, approved on 14 May 2023). The study was conducted on a research farm located in Gyeongsangbuk-do, South Korea, under standardized management conditions. A total of 107 Hanwoo cows were included in the initial selection based on their reproductive status, with estrus synchronization achieved using the GnRH-based OvSynch Protocol [32]. Artificial insemination was performed during May and June 2023 to ensure uniformity in reproductive timing. Real-time ultrasonography was used to confirm pregnancy and assess fetal sex during the gestation period. Out of the initial 107 cows, 3 were excluded from further analysis due to inconclusive ultrasound readings, resulting in a final sample size of 104 cows for detailed examination. Ultrasonographic fetal sex determination was conducted at three gestational stages: 55–70 days, 71–85 days, and 86–100 days. Each cow underwent ultrasound examinations at these stages to monitor pregnancy status and identify fetal sex, ensuring comprehensive data collection throughout the study period. The pregnant Hanwoo cows exhibited a normal ovarian cycle and were 4.1 ± 0.6 (mean ± SEM) years of age. They were maintained under uniform housing, feeding, and veterinary protocols to minimize variability. The mean Body Condition Score (BCS) of the cows was 3.0 ± 0.2 on a scale of 1 to 5, where 1 indicates a very thin condition and 5 indicates a very fat condition [33,34]. This standardized management approach ensured consistency across all experimental conditions, enabling reliable comparisons of ultrasonographic fetal sex determination across the different gestational stages.

### 2.2. Ultrasound Scanner Examination

An ultrasound examination was performed using a B-mode real-time ultrasound scanner (Easi-Scan Go bovine ultrasound scanner, IMV Technologies, L’Aigle, France) operating at a frequency of 5–7.5 MHz to diagnose pregnancy in cows between 55 and 100 days after artificial insemination. Transrectal ultrasound was performed according to the position of the fetus, with the transducer inserted through either the right or left uterine horn, using both longitudinal and transverse views. Each cow underwent only one scan, and the procedure was performed by an experienced technician to ensure consistency and accuracy of results [24]. The duration of each examination was restricted to 5 min or less.

### 2.3. Fetal Sex Determination

The sex of the fetus was identified by observing specific anatomical structures. For male fetuses, the presence of a scrotum located between the hind legs or a genital tubercle (penis or prepuce) positioned directly behind the umbilicus was used as a definitive indicator of male sex. The genital tubercle was typically detected earlier in gestation, appearing as a distinct protrusion around 55–70 days, while the scrotum became more readily observable in later stages, particularly after 70 days, as fetal development progressed.

For female fetuses, the presence of mammary tissue or a genital tubercle (vulva) located beneath the tail was used to determine sex. Mammary tissue appears as a small circular or oval shape, while the vulva is seen as a flat protrusion in the perineal area. These structures are distinguishable from male genitalia based on their shape and location. The vulva and mammary glands were consistently detectable from 55 days onward, with optimal visibility achieved between 71 and 85 days when fetal positioning allowed clearer imaging.

The ultrasound findings for each fetus were recorded during the examination. The fetal sex, as determined by ultrasound, was documented and later verified at the time of birth to assess the accuracy of the prenatal diagnosis. The diagnostic outcomes were categorized as either “correct”, “incorrect”, or “inconclusive” based on the comparison between the recorded fetal sex and the actual sex of the newborn calf. The accuracy rate, error rate, and rate of inconclusive diagnoses were calculated for each stage of gestation (55–70 days, 71–85 days, and 86–100 days) to evaluate the reliability of the ultrasound technique at different stages of pregnancy [35].

### 2.4. Economic Analysis

#### 2.4.1. Farm Management Scenarios

The economic analysis was conducted to evaluate the financial impact of using fetal sex determination technology in Hanwoo cattle under different farm management strategies [36]. The analysis was performed using data obtained from the actual market values of breeding cows and calves, and it considered both breeding and purchasing scenarios.

In the first scenario, the focus is on maximizing profit for the breeding farm by setting the price of pregnant cows based on the expected sex of the calf. Since male calves generally have a higher market value compared to female calves, the breeding farm can set a premium price for cows carrying male fetuses. This scenario assumes that the breeding farm has access to reliable fetal sex determination data and uses this information to differentiate the pricing of pregnant cows. By selling cows carrying male fetuses at a higher price, the breeding farm aims to increase its total revenue and profit margin. This strategy, while beneficial for the breeding farm, may reduce the profit potential for purchasing farms that buy pregnant cows without prior knowledge of the calf’s sex, as they will have to pay a higher price for cows carrying male fetuses without a corresponding increase in revenue.

The second scenario explores the strategy of minimizing costs for the purchasing farm. Given the price difference between male and female calves, the purchasing farm aims to negotiate lower prices for cows carrying female fetuses, which have a lower market value. In this scenario, the price of cows carrying male fetuses is maintained at the standard market rate, while the price of cows carrying female fetuses is reduced to reflect their lower expected income. This approach allows the purchasing farm to save on acquisition costs when buying cows carrying female fetuses. Although the income from selling female calves remains lower than that of male calves, the reduced initial cost can help the purchasing farm improve overall profitability by minimizing upfront expenses. This scenario evaluates the potential financial benefits of cost-saving strategies for purchasing farms that have access to fetal sex determination information.

#### 2.4.2. Variables and Fixed Assumptions in Economic Analysis

The economic analysis of fetal sex determination in Hanwoo cattle was conducted using a set of predefined variables and assumptions to ensure consistency and reliability in the evaluation. The key variables incorporated in the analysis included market prices, breeding cow values, calf values, ultrasound examination costs, raising costs, and the sex ratio of the calves. The market prices used in the study were based on the national average transaction prices for breeding cows and calves as of July 2024. These prices provided a reference point for determining the economic value of both male and female calves and were applied consistently across all scenarios.

The breeding cow value was assumed to be constant for all cases, regardless of whether the calf was male or female, to focus on the variation in calf prices as a determinant of profitability. Calf value was identified as a crucial factor, as the market value of male calves is typically higher than that of female calves due to the greater demand for male calves in meat production. This price differential was incorporated into the analysis to reflect the actual market conditions. The analysis also took into consideration the cost associated with performing ultrasound examinations. The expenses included the cost of the ultrasound equipment, technician labor, and other operational costs. These costs were considered as part of the total expenses incurred by the breeding farm in scenarios utilizing fetal sex determination. Raising costs were assumed to be equivalent for both male and female calves, as it is generally accepted that the costs of feed, veterinary care, and labor do not differ significantly between sexes. This assumption simplified the analysis by ensuring that any variation in profitability was attributed solely to differences in market prices and not to discrepancies in raising costs. A 1:1 sex ratio was assumed for male and female calves, which is the expected natural distribution in cattle populations. This ratio was applied to predict the outcomes in scenarios without fetal sex determination, where the probability of acquiring a male or female calf was equal. In scenarios involving fetal sex determination, the known sex of the fetus allowed for more precise planning and strategy development by the farm. The assumptions outlined above ensured that the analysis was conducted under controlled and consistent conditions, allowing for a clear comparison between the economic outcomes of different management strategies. These assumptions also provided a realistic representation of market conditions and farm operations, supporting the validity and applicability of the results in real-world settings.

### 2.5. Calculation of Economic Metrics

The economic impact of utilizing fetal sex determination technology in Hanwoo cattle was evaluated using several key economic metrics [37]. These metrics were carefully selected to provide a comprehensive understanding of profitability, cost-effectiveness, and the overall financial benefits of the technology under different management scenarios. Total revenue represents the sum of the value of the breeding cow and the calf, providing a baseline measure of financial returns under each scenario [36]. The total revenue is then compared across the different scenarios to evaluate how fetal sex determination and strategic management decisions influence the revenue generated by the farm.

The formula used to calculate total revenue is as follows:

*R* = Breeding Cow Value + Calf Value

For scenarios involving male calves, the total revenue is calculated as follows:

*R*_male_ = Breeding Cow Value + Male Calf Value

For scenarios involving female calves, the formula is as follows:

*R*_female_ = Breeding Cow Value + Female Calf Value

Profit is calculated as the difference between total revenue and total costs. It serves as a primary indicator of financial performance, capturing the net economic benefit after accounting for all expenditures associated with each scenario [38]. Total costs include not only the cost of purchasing and raising the breeding cow but also any additional costs incurred for fetal sex determination, such as the cost of ultrasound examination, labor, and other related expenses. By comparing the profit values across different scenarios, the study evaluates the effectiveness of fetal sex determination in enhancing overall farm profitability.

The formula used for profit calculation is as follows:

*P = R* − Total Costs

Net profit margin is used to measure the profitability of each scenario relative to the total revenue generated. It provides insights into the efficiency of resource utilization and the proportion of revenue that translates into profit. The net profit margin allows for a direct comparison of profitability across different scenarios, enabling an assessment of which management strategy yields the highest return on investment.

The net profit margin is calculated as follows:

$$\mathrm{NPM}=\frac{P}{R}\times 100$$

The breakeven point analysis identifies the minimum revenue required to cover all costs, providing a measure of the financial feasibility of using fetal sex determination technology [39]. This analysis helps determine the conditions under which the technology would be economically viable, considering variables such as market prices, calf values, and the costs associated with implementing the technology. By establishing the breakeven point for each scenario, the study assesses the financial risks and potential profitability associated with using fetal sex determination technology under varying market conditions.

The breakeven point is calculated as follows:

$$\mathrm{Breakeven}\;\mathrm{Point}=\frac{Revenue\;per\;Unit}{Total\;Costs}$$

A cost–benefit analysis was conducted to evaluate the overall economic benefit of using fetal sex determination technology [40]. The analysis compares the total costs incurred (e.g., cost of ultrasound, labor) with the financial returns (total revenue) for each scenario. A benefit–cost ratio (BCR) greater than 1 indicates a positive return on investment, suggesting that the economic benefits outweigh the costs. Conversely, a BCR less than 1 suggests that the costs exceed the benefits, indicating a negative return on investment. This metric provides a clear assessment of the financial value of adopting fetal sex determination technology.

The BCR is used as a key metric to quantify the return on investment:

$$\mathrm{BCR}=\frac{Total\;Costs}{Total\;Benefits}$$

### 2.6. Statistical Analysis

The data were analyzed using descriptive statistics to evaluate the economic performance of each scenario. Profitability and cost-effectiveness were compared across scenarios, and sensitivity analysis was conducted to identify key variables that influenced economic outcomes [41]. Statistical significance was determined using Analysis of Variance (ANOVA) to compare the mean profitability between scenarios, with a significance level set at *p* < 0.05. A sensitivity analysis was performed to assess how changes in key variables, such as calf prices, ultrasound costs, and sex ratio, impacted profitability and the overall economic outcome. This analysis provided insights into the robustness of each scenario under varying market conditions.

## 3. Results

This section may be divided by subheadings. It should provide a concise and precise description of the experimental results and their interpretation, as well as the experimental conclusions that can be drawn.

### 3.1. Overall Detection Rate

**Figure 1:**
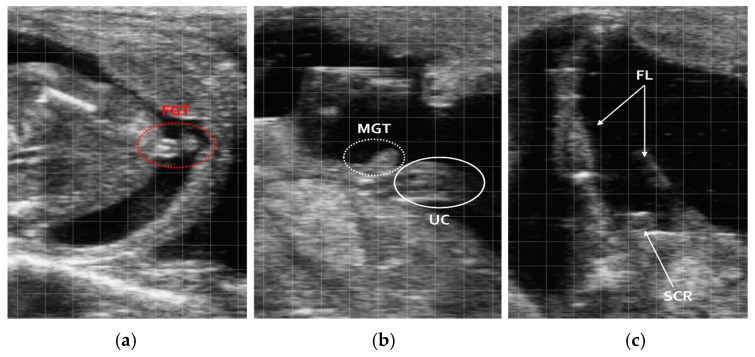
Key anatomical markers for fetal sex determination in Hanwoo cows using ultrasound at 86–100 days of gestation. (**a**) Female; (**b**) male; (**c**) female genital tubercle (FGT); male genital tubercle (MGT); umbilical cord (UC); fore limbs (FLs); scrotum (SCR).

Using ultrasonography, fetal sex was successfully determined in 104 out of 107 animals (96.2%), with 3 cases (3.7%) yielding inconclusive results. Ultrasound examination provided distinct anatomical markers for each sex: in male fetuses, the genital tubercle was located caudally near the umbilicus, with the scrotum positioned between the legs and near the overdeveloped ridge. In female fetuses, the genital tubercle was positioned below the tail, and mammary glands were observed in a parallel orientation close to the midline (Figure 1).

### 3.2. Accuracy of Sex Determination Across Gestational Periods

The accuracy of fetal sex determination was assessed at three gestational stages, as summarized in Table 1. Each animal underwent ultrasound examinations at these stages, and the results were later verified against the actual sex at birth to validate the diagnostic accuracy of the method.

**Figure 2:**
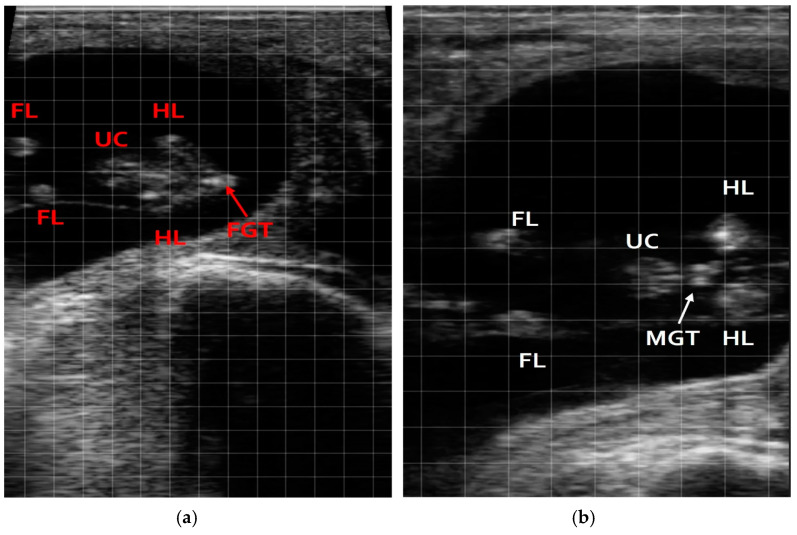
Still image ultrasound of a fetus between 50 and 70 days of pregnancy. (**a**) Female; (**b**) male, hind limbs (HLs); fore limbs (FLs); umbilical cord (UC); male genital tubercle (MGT); female genital tubercle (FGT).

In the first gestational period (55–70 days), ultrasound correctly identified 104 fetuses as 52 males and 52 females. Following birth, these identifications were confirmed, with slight variance (53 males, 51 females), resulting in an accuracy rate of 98.0% (Figure 2).

**Figure 3:**
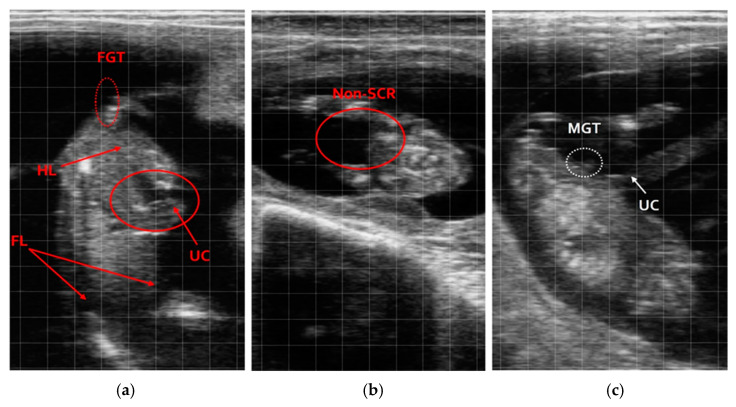
Still image ultrasound of a fetus between 71 and 85 days of pregnancy. (**a**) Female aerial view; (**b**) female posterior view; (**c**) male, hind limbs (HLs); fore limbs (FLs); umbilical cord (UC); male genital tubercle (MGT); female genital tubercle (FGT); scrotum (SCR).

In the second gestational period (71–85 days), both male (penis and scrotum) and female (vulva and tail) anatomical features were clearly visible. This clarity allowed for fast and highly accurate identifications, achieving a 100% accuracy rate, as all 104 animals were correctly identified as 53 males and 51 females, fully matching the birth outcomes (Figure 3).

**Figure 4:**
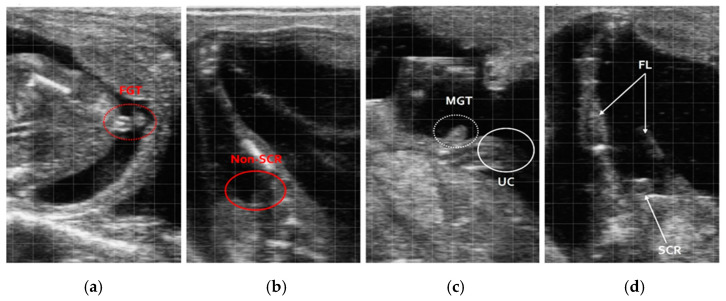
Still image ultrasound of a fetus between 86 and 100 days of pregnancy. (**a**) Female aerial view; (**b**) female posterior view; (**c**) male aerial view; (**d**) male posterior view; fore limbs (FLs); umbilical cord (UC); male genital tubercle (MGT); female genital tubercle (FGT); scrotum (SCR).

The precision observed in this period is reflected in Table 2, demonstrating that this gestational window provides optimal conditions for accurate fetal sexing. During the third gestational period (86–100 days), although ultrasound images showed clear anatomical structures, identification became more challenging due to the presence of uterine cotyledons and the higher BCS typical of Hanwoo cows. Despite these limitations, ultrasound still achieved a high accuracy rate of 98.0%, with 52 males and 52 females identified by ultrasound and later verified as 53 males and 51 females at birth (Figure 4).

Table 1 consolidates these findings, showing no statistically significant differences in accuracy across the three gestational periods (*p* > 0.05). This consistency in detection rates underscores the reliability of ultrasonography for fetal sex determination across all stages of gestation in Hanwoo cows. The strong alignment between ultrasound results and actual birth outcomes further validates the accuracy and practical applicability of this technique in field settings.

### 3.3. Economic Impact of Fetal Sex Determination in Pregnant Hanwoo Cows

The current market value for pregnant Hanwoo cows, without fetal sex determination, reflects an average calf value of USD 2000, calculated based on the average price of male and female calves. As a result, the total asset value for both breeding and purchasing farms stands at USD 4340. Table 3 represents the current state of the market, where fetal sex is not considered during transactions, leading to a uniform valuation for all pregnant cows regardless of the eventual calf sex. In the scenario where the calf is female, the total asset value for the breeding farm is USD 3960 (USD 2340 for the breeding cow and USD 1610 for the female calf). If the calf is male, the total asset value rises to USD 4720 (USD 2340 for the breeding cow and USD 2370 for the male calf). The difference in asset value between a breeding farm with a male calf and one with a female calf is USD 760, highlighting the potential economic impact of incorporating fetal sex determination in farm management decisions (Table 2).

Two distinct pricing scenarios are possible. In the first scenario, the breeding farm selling the pregnant cow aims to maximize profit, setting the price of a cow pregnant with a female calf at USD 3850 and increasing the price of a cow pregnant with a male calf to USD 4610 (USD 3840 + USD 760) (Table 3). This strategy yields the greatest profit for the breeding farm but reduces the profit potential for the purchasing farm, which acquires the cow without knowing the calf’s sex. The income received by the purchasing farm would remain the same, regardless of the calf’s sex. In the second scenario, the purchasing farm aims to minimize costs. Given the price difference between male and female calves in the market, the purchasing farm would seek to lower the price for cows pregnant with female calves. In this case, the price of a cow pregnant with a male calf remains USD 3850, while the price of a cow pregnant with a female calf is reduced to USD 3090 (USD 3850 − USD 760) (Table 4). This results in the purchasing farm saving costs when acquiring a cow pregnant with a female calf, although the income remains unchanged.

## 4. Discussion

In this study, fetal sex determination in Hanwoo cows via ultrasonography demonstrated both promising potential and inherent challenges, with an overall accuracy rate of 96.2% achieved across 107 cases. The effectiveness of fetal sex identification varied across gestational stages due to anatomical visibility and physiological characteristics specific to the Hanwoo breed, which, as a meat-focused breed, typically displays greater fat deposition compared to dairy breeds like Holstein. This higher BCS made identification particularly challenging in later gestational stages, as did the encroachment of cotyledon and muscle tissues on the fetal imaging field. Compared to studies on Holstein or Angus breeds, which report varying levels of success with ultrasonographic sex determination and economic feasibility, this study underscores the specific challenges and benefits associated with Hanwoo farming. For instance, the higher BCS of Hanwoo cows significantly impacts the visibility of anatomical markers, which is less frequently addressed in studies focusing on dairy cattle. During the 55–70-day period, focusing on female fetuses proved beneficial, as the vulva and tail intersection in females became more visible, allowing ultrasound interpreters greater confidence in their observations. At 71–85 days, anatomical markers in both male and female fetuses, such as the penis, scrotum, vulva, and tail, were markedly clearer, enabling faster and more intuitive evaluations, which helped achieve the highest diagnostic accuracy during this period [31]. However, in the 86–100 day range, the increased BCS in Hanwoo cows and the larger presence of uterine cotyledons significantly obstructed views, making accurate identification more challenging [28]. Ultrasound evaluation in later gestation stages (beyond 110 days) was notably limited by the positioning of the fetus deep within the abdominal cavity, which further obstructed key anatomical markers essential for fetal sexing. This finding aligns with prior research [27,42], which indicates that as pregnancy progresses, anatomical markers such as the scrotum and genital tubercle become harder to observe due to the fetus’s deeper positioning. These observations suggest that, while ultrasonography remains a valuable tool for fetal sex determination in Hanwoo cows, optimal results are obtained when examinations are conducted in earlier stages of pregnancy, where anatomical visibility is highest.

The experience of the technician is another variable that has a significant impact on the accuracy of the diagnosis. Studies have demonstrated that technicians with greater experience are better able to navigate the challenges posed by fetal positioning and anatomical complexity, resulting in higher accuracy rates [24,26]. In this study, the technician was experienced; however, the constraints of a single scan per animal may have limited the overall success rate. Prior research has shown that technicians who refine their skills through repeated scans, sometimes in abattoirs or other controlled environments, can markedly enhance the accuracy of their diagnoses [28]. This indicates that with more comprehensive training and multiple scans, the accuracy rates in this study could potentially be on par with or exceed the 96.3% accuracy reported in other studies [31].

Moreover, it is imperative to consider the economic implications of fetal sex determination in future applications (Table S1). From an industry-level perspective, the determination of the sex of an animal presents both opportunities and challenges. One of the principal advantages is the capacity to differentiate prices in accordance with the anticipated sex of the calf, which has the potential to enhance profitability for breeding farms [6,8]. In this study, it was estimated that a male calf would result in an increase of approximately USD 760 in the overall asset value of the farmer compared to a female calf. For a breeding farm, this creates an opportunity to maximize profit by adjusting the price of cows based on fetal sex, such as selling cows carrying male calves at a premium (USD 4613 for male calves vs. USD 3850 for female calves).

Nevertheless, this strategy also presents certain difficulties, particularly for purchasing farms. Should breeding farms implement price increases for cows based on the sex of the calf, purchasing farms, especially those engaged in beef production, may encounter diminished profit margins. For instance, a purchasing farm that pays a higher price for a cow carrying a male calf may not see a corresponding increase in profit if market prices adjust. Furthermore, there remains a small margin of error in fetal sex determination, and a situation where a farm purchases a cow with a male fetus but the born calf turns out to be female could lead to financial loss. Such discrepancies might necessitate reassessments of pricing strategies or contractual agreements between breeding and purchasing farms to mitigate risk. For example, purchase contracts could include clauses for compensation or price adjustments in cases where the calf’s sex does not match the fetal sex determined during ultrasonography. Addressing these potential outcomes in economic models is crucial for fostering fairer transactions and minimizing economic risks for purchasing farms.

Conversely, the ability to determine the sex of an animal could enable purchasing farms to reduce costs by negotiating lower prices for cows carrying female calves. In the second scenario examined in this study, the price of a cow carrying a female calf could decline to USD 3087, while the price of a cow carrying a male calf would remain at USD 3850. This price differentiation could render the purchase of female calves more appealing for certain operations, such as those focused on dairy or breeding purposes. Furthermore, it could contribute to a more balanced market, wherein both male and female calves offer distinct economic advantages [26,27].

However, it is essential to consider the financial implications of implementing sex determination technology. The costs associated with fetal sex determination, estimated at USD 140 per cow, must be considered in the broader economic context [31]. For large-scale operations involving thousands of cows, these costs could be significant, potentially negating the benefits gained through price differentiation. In a hypothetical scenario involving 100,000 pregnant cows, the total cost of performing sex determination would be USD 14 million. Nevertheless, should the price differential between male and female calves remain constant, the additional revenue generated could reach USD 38 million, resulting in a net benefit of USD 24 million for the industry. These figures indicate that although the initial costs of implementing sex determination technology may be considerable, the long-term financial benefits could be significant, particularly for breeding farms that can capitalize on price differentiation.

Furthermore, market dynamics are of great consequence in determining the long-term viability of sex determination in the cattle industry. Widespread adoption of this technology could result in an oversupply of either male or female calves, depending on market preferences [8]. This could subsequently lead to a decline in prices and a reduction in economic benefit. For example, if sex determination results in an oversupply of male calves due to their higher perceived value, the price of male calves could decline, thereby reducing the financial advantage for farms that have invested in sex determination. Conversely, should female calves become the preferred option for breeding or dairy operations, a comparable oversupply could ensue, potentially affecting profitability.

In addition to shifts in market supply and demand, the long-term sustainability of sex determination technology will depend on the extent to which the industry can adapt to its widespread use. Integrating sex determination into broader operational strategies, such as adjusting breeding practices or marketing efforts, may confer a competitive advantage to farms that are able to achieve this [36]. Conversely, farms that do not adopt sex determination may encounter difficulties in maintaining competitiveness, particularly if market prices begin to reflect the growing use of this technology.

While ultrasonographic sex determination has been widely studied in dairy and large-scale cattle production systems, its application in Hanwoo farming provides a new perspective on integrating advanced reproductive technologies with economic decision-making. The results demonstrate how this technology can alleviate profitability gaps caused by calf sex preferences, a challenge not limited to Hanwoo cattle but relevant to other small-scale beef cattle operations globally. This study’s findings pave the way for further research on combining sexed sperm and ultrasonography as part of a holistic approach to sustainable livestock management.

## 5. Conclusions

In conclusion, the determination of fetal sex in Hanwoo cows has the potential to offer significant economic benefits, particularly for breeding farms. The capacity to differentiate prices based on calf sex can enhance profitability, while purchasing farms could benefit from more informed purchasing decisions. Nevertheless, the ultimate impact on the industry will be contingent upon a multitude of variables, including the experience of the technicians involved, prevailing market dynamics, and the financial implications associated with implementing the technology. As the cattle industry continues to evolve, fetal sex determination may become an increasingly important tool for optimizing both breeding and purchasing strategies. However, its long-term success will require careful consideration of its economic and practical implications across the entire supply chain.

## Figures and Tables

**Table 1:** Comparison of fetal sex determination accuracy and actual birth sex across three examination stages.

Examination Stage	No. of Animals (*n*)	Males (*n*)	Females (*n*)	Correct Diagnoses (%)
1st diagnoses (55–70 days)	104	52	52	98.0
2nd diagnoses (71–85 days)		53	51	100
3rd diagnoses (86–100 days)		52	52	98.0
Post-birth		53	51	-

The Chi-Square test results indicate that there is no statistically significant difference (*p* > 0.05) between the fetal sex determined by ultrasound and the actual sex observed at birth for each of the three examination stages.

**Table 2:** Economic analysis of market value for pregnant Hanwoo cows based on fetal sex determination.

Breeding Farm	Breeding Cow Value (USD)	Calf Value (USD)	Total Asset Value (USD)
Female calf	2340	1610	3960 ^a^
Male calf		2370	4720 ^b^
Current market case (Average)		2000	4340 ^ab^

The total asset value for breeding farms with male calves (USD 4720) was significantly higher compared to farms with female calves (USD 3960), as indicated by different letters (a, b) in the table (= *p* < 0.05). The current market scenario, which does not incorporate fetal sex determination and assumes an average calf value of 2.86 million KRW, shows an intermediate total asset value (USD 4720) that did not differ significantly from either the male or female scenarios, as indicated by the shared letter (ab).

**Table 3:** Economic analysis of fetal sex determination in the context of maximizing profit for the breeding farm.

		Breeding Cow Value (USD)	Calf Value (USD)	Total Asset Value (USD)
Breeding Farm	Female Calf	2330	1617	3955
	Male calf		2380	4718 *
Purchasing Farm	Female Calf		1617	3850
	Male calf		2380	4610 *
Price Difference	Male–Female	-	760	760

Economic analysis of fetal sex determination, demonstrating profit maximization strategies for breeding farms, with a statistically significant difference (*p* < 0.05 *) in total asset value between male and female calf scenarios.

**Table 4:** Economic analysis of fetal sex determination in the context of minimizing costs for the purchasing farm.

		Breeding Cow Value (USD)	Calf Value (USD)	Total Asset Value (USD)
Breeding Farm	Female Calf	2330	1617	3955
	Male calf		2380	4718 *
Purchasing Farm	Female Calf		1617	3087
	Male calf		2380	3850 *
Price Difference	Male–Female	-	760	760

Economic analysis of fetal sex determination, illustrating cost minimization strategies for purchasing farms, with a statistically significant difference (*p* < 0.05 *) in total asset value between male and female calf scenarios.

## Data Availability

The data presented in this study are available upon request from the corresponding author.

## Supplementary Materials

The following supporting information can be downloaded at https://www.mdpi.com/article/10.3390/vetsci12030201/s1: Table S1: Economic analysis model design: comparison of three scenarios.
