# Supplementary material for: Economic Impacts of Ultrasonographic Fetal Sex Determination on Hanwoo Cattle Profitability and Market Dynamics
Source: Vet Sci. 2025 Feb 27;12(3):201. doi: 10.3390/vetsci12030201 (PMC11946265; doi:10.3390/vetsci12030201)
Supplement: Supplementary file 1 [file vetsci-12-00201-s001.zip › vetsci-3440430-supplementary.pdf]

Table S1. Economic Analysis Model Design: Comparison of Three Scenarios

Scenario	Description	Key Variables	Expected Outcome
Without Fetal Sex Determination	Standard market price is used for cows without knowing the calf's sex, assessing risk and profitability without fetal sex determination.	Breeding Cow Value, Calf Value, Standard Market Price.	Higher risk and uncertainty in profitability due to unknown calf sex.
Maximizing Profit for Breeding Farm	Breeding farm sets the price of pregnant cows based on the expected sex of the calf to maximize profit.	Breeding Cow Value, Calf Value, Price Difference, Profit Margin.	Increased revenue for the breeding farm but reduced profit potential for the purchasing farm.
Minimizing Costs for Purchasing Farm	Purchasing farm negotiates prices based on the expected lower revenue from female calves to minimize costs.	Breeding Cow Value, Reduced Price for Female Calves, Savings.	Lower acquisition costs for the purchasing farm, potential savings based on female calf purchases.
